# Botulinum toxin type A with or without needle electromyographic guidance in patients with cervical dystonia

**DOI:** 10.1186/s40064-016-2967-x

**Published:** 2016-08-08

**Authors:** Chuanjie Wu, Fang Xue, Wansheng Chang, Yajun Lian, Yake Zheng, Nanchang Xie, Lu Zhang, Chen Chen

**Affiliations:** 1 Department of Neurology, The First Affiliated Hospital of Zhengzhou University, Zhengzhou, 450052 China; 2 Department of Neurology, The Second Hospital of Hebei Medical University, Shi Jiazhuang, China; 3 Department of Neurology, The Second People’s Hospital of Liaocheng, Shandong, China

**Keywords:** Cervical dystonia, Botulinum toxin type A, Electromyography

## Abstract

**Aim:**

To investigate the efficacy and safety of electromyography (EMG)- and palpation-guided botulinum toxin type A injection in cervical dystonia (CD) patients.

**Methods:**

In this randomized, controlled trial, 68 CD patients were randomly allocated to two groups, receiving botulinum toxin type A injections guided by either palpation (Group A) or EMG (Group B). The primary endpoint is defined as the difference in the Tsui score between groups at 16 weeks. The secondary endpoints were the visual analog scale (VAS) and Hospital Anxiety and Depression Scale (HADS) scores and Clinical and Patient Global Impression of Change (CGIC and PGIC).

**Results:**

Sixty-five patients completed the study. No significant difference was observed in the Tsui score between groups A and B at 4, 8, and 12 weeks after treatment (p > 0.05). However, 16 weeks after treatment, the Tsui score of group A was significantly higher than that of group B. For both groups, the degree of pain at each time point during follow-up significantly reduced after treatment. However, no significant difference was observed in VAS scores between the two groups. Interestingly, the patient HADS score decreased without statistical significance 8 weeks following treatment. No significant difference in HADS scores was observed between the two groups. Additionally, there was no significant difference in PGIC and CGIC between the two groups. However, CGIC was significantly higher than PGIC. No significant difference in adverse reactions was observed between groups. CD patients treated with EMG guidance experienced a significantly more pain at the injection site but a significantly lower adverse event occurrence rate of dysphagia when compared to CD patients treated with palpation guidance only.

**Conclusions:**

CD patients treated with EMG guidance experienced a prolonged benefit as measured by the Tsui scale when compared to CD patients treated with palpation guidance alone. EMG-guided injection resulted in a lower incidence of dysphagia and higher incidence of discomfort at the injection site than palpation-guided injection.

## Background

Cervical dystonia (CD) is the most common form of focal dystonia. It is characterized by involuntary muscular contractions resulting in abnormal neck and shoulder movements and postures, which can involve tremor and pain. This disorder is profoundly distressing and can negatively impact the patient’s quality-of-life (Comella and Bhatia 2015). CD is more common in women than in men and occurs in 28–183 individuals per 100,000 (Defazio et al. 2013).

Botulinum toxin type A is a serotype (A, B, C1, C2, D, E, F, and G) of botulinum neurotoxin derived from *Clostridium botulinum* (Setler 2002). It was first used clinically in the 1980s for the treatment of strabismus. Since its introduction, botulinum toxin type A has been used for a variety of clinical conditions, including hemifacial spasm, Meige’s syndrome, migraine, and CD. Intramuscular injection of botulinum toxin type A is effective and well-tolerated for CD treatment (Truong et al. 2005) and has therefore been recommended as a first-line therapy under current treatment guidelines (Albanese et al. 2011; Simpson et al. 2016).

Numerous studies have focused on the optimal dose, short-term and long-term efficacies, and botulinum toxin (BT) serotype efficacy for the treatment of CD (Pappert et al. 2008; Dressler et al. 2013; Poewe et al. 1998). However, few studies have evaluated BT injection technique for the treatment of CD (Simpson et al. 2016; Walker et al. 2015; Comella et al. 1992). Currently, most clinical practices inject BT using palpation for guidance. Other commonly used injection techniques include electromyography (EMG), electrical stimulation, or ultrasound guidance (Walker et al. 2015). Therefore, we compared the efficacy and safety of EMG- and palpation-guided botulinum toxin type A injection in CD patients.

## Methods

### Study design

This is a prospective, randomized, single-blind, clinical trial evaluating response of CD treated with botulinum toxin A administered using EMG guidance versus palpation. The ethics review boards approved the study protocol. The goal, procedure, and safety aspects of the study were explained to each patient. All patients provided written informed consent to participate in the study.

All treatments were administered by a neurologist who did not participate in the randomization and investigation procedures. Physician raters were blinded to treatment assignment. After obtaining baseline measurements, patients meeting the study criteria were randomly divided into two groups. Group A received botulinum toxin type A injections guided by palpation, whereas group B received botulinum toxin type A injections guided by EMG. We allocated patients to one of the two groups using a restricted randomization scheme generated by SPSS. The overall duration of the study for each patient was 16 weeks, and follow-up visits were conducted every 4 weeks after the injection. Patients were free to discontinue the trial at any time during the study. Patients who were on antispasmodic and/or antidepressant medications were requested to remain on baseline medication doses for the duration of the study.

### Study participants

Patients were recruited at the First Affiliated Hospital of Zhengzhou University, the Second Hospital of Hebei University and the Second People’s Hospitla of Liaocheng between February 2014 and March 2015. Criteria for entry into the study were: (1) patients aged between 18 and 80 years, (2) primary focal CD without other dystonia (diagnosis made by clinical symptoms and EMG), (3) CD history ≥6 months, (4) no previous history of botulinum toxin therapy or a minimum interval of 18 weeks since the last injection, and (5) TSUI score ≥9 at baseline.

Exclusion criteria included patients with any medical condition or use of any agent that might put them at increased risk if exposed to botulinum toxin type A (e.g. neuromuscular disorders or agents that might interfere with neuromuscular function), infections or skin problems at the injection site, patients who weighed less than 45 kg, or had pure anterocollis. Women of childbearing age were required to have a negative pregnancy test result prior to injection. Furthermore, women who were pregnant, nursing, or planning a pregnancy during the study, or who were unable or unwilling to use a reliable form of contraception during the study were also excluded.

### Treatments

All treatments were administered in the treatment room of the Department of Neurology, which is equipped with all the necessary facilities in case of severe reactions or emergencies.

Botulinum toxin type A (100 U of *C. botulinum* type A neurotoxin complex, 5 mg gelatin, 25 mg dextran, and 25 mg saccharose) was obtained from Lanzhou Biological Products Institute (Hengli, Lanzhou, China). Each vial was diluted in 2 mL of saline solution (0.9 %) as recommended by the manufacturer. Injection doses for each muscle were as follows: 25–100 U for the sternocleidomastoid muscle, 50–100 U for the splenius capitis muscle, 25–100 U for the trapezius muscle, 25–60 U for the levator scapulae, and 10–25 U for the anterior, middle and posterior scalene muscle. The specific injection dose for each muscle was determined based on clinical conditions and controlled within the above ranges by the physician administering the injections.

In the group receiving injection guided by palpation, the physician identified a bony landmark and used palpation to identify the target muscles. Target muscles were selected based on the following aspects: (1) posture of the head and the neck at rest and during passive and voluntary movement, as well as biomechanical analysis of involuntary movement; (2) the location of neck pain or traction at rest and during passive and voluntary movement; (3) and the site of hypertrophy or stiffness of the neck muscles identified by observation or palpation. The injections were administered using a 1-mL syringe with a 0.45-mm needle.

In the group receiving injection guided by EMG, a special EMG electrode with a hollow core and insulated exterior was employed, with the recording lead connected to the EMG recorder and the tail end connected to the injection syringe. Injection sites were selected based on the following aspects: (1) spontaneous tonic activity or muscle electrical activity in quartiles at rest; (2) spontaneous low to median frequency (4–9 Hz) tremor-type electrical activity; (3) presence of excitation interference by antagonists on voluntary movements; (4) and sensory maneuvers that can reduce EMG activity in dystonia.

### Efficacy and safety measures

The primary endpoint of the study was the Tsui score at 16 weeks (Tsui et al. 1986). The Tsui score was assessed using standardized conditions (at least 1-min assessment with the patient sitting in a comfortable chair, eyes open, making a subjective effort to relax the head and neck, and refraining from sensory maneuvers during the assessment).

The secondary endpoints were head and neck region pain as assessed based on the visual analog scale (VAS) score every 4 weeks, symptoms of anxiety and depression as assessed by the Hospital Anxiety and Depression Scale (HADS) at 8 weeks, and overall response to treatment as assessed by the Clinical and Patient Global Impression of Change (CGIC and PGIC, respectively) scale at 16 weeks. HADS is a self-assessment scale for evaluating the current severity of depression and anxiety. A HADS score >7 represents the presence of anxiety or depression. Higher scores reflect marked impairment. PGIC is a self-evaluation of the patient’s overall change since the start of the study on a 7-point scale (1, very much improved; 2, much improved; 3, minimally improved; 4, no change; 5, minimally worse; 6, much worse; 7, very much worse), and was obtained at the end of the study.

Safety was evaluated based on the occurrence of adverse reactions. Adverse events were assessed at each clinic visit using a combination of open questioning and a checklist of ten conditions (Wissel et al. 2001) (dysphagia, dry mouth, voice changes, neck muscle weakness, tiredness, respiratory difficulties, discomfort at injection site, visual difficulties, jaw weakness, and limb weakness) commonly considered to be related to botulinum toxin type A use. Once an adverse reaction occurred, the time of the adverse reaction occurrence, its duration time, the occurrence frequency, severity, whether treatment was required, and the treatment outcome were recorded.

### Statistical analysis

The sample size was estimated from treatment differences using similar previous CD studies (Comella et al. 1992). This sample size would provide 80 % power at two-sided significance level of 0.05.

All analyses were performed on the intent-to-treat (ITT) population, and all statistical testing was two-sided. The quantitative data were assessed using the mean and standard deviation. The *t* test was performed to compare the age, duration of disease, Tsui score between groups at 4, 8, 12 and 16 weeks, VAS every 4 weeks, and HADS score between the two groups at 8 weeks. The Chi square test was performed to assess differences in gender, PGIC and CGIC distribution at 16 weeks, and the proportion of adverse reactions between two groups. The SPSS 13.0 software package was used for statistical evaluation, and a p value <0.05 was considered statistically significant.

## Results

### Patient characteristics

**Fig. 1:**
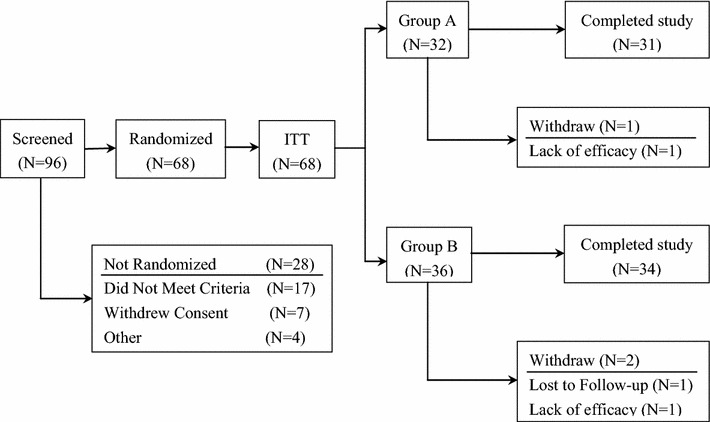
Flow diagram showing progression of subjects from screening

The recruitment period was between February 2014 and March 2015, with a 16-week follow-up period after the last patient was enrolled. Ninety-six patients were screened, and 68 were randomized (32 guided by palpation, 36 guided by EMG). Sixty-five patients completed the study. A flow diagram of the study is shown in Fig. 1. There were no significant between-group differences in demographic data, baseline clinical characteristics, or botulinum toxin type A dose (Table 1).

**Table 1:** Demographic and baseline clinical characteristics

Parameter	Group A	Group B	Total
Age (years)			
Mean (SD)	44.5 (11.0)	43.1(10.7)	43.7 (10.7)
Median	46	42	43.5
Min/max	21/66	26/69	21/69
Female, N (%)	22 (68.8 %)	23 (63.8 %)	46 (66.2 %)
Weight (kg)			
Mean (SD)	64.7 (6.9)	65.8 (7.8)	65.3 (7.3)
Median	65	65.5	65.5
Min/max	49/80	51/81	49/81
CD duration (years)			
Mean (SD)	6.0 (2.5)	5.8 (2.0)	6.0 (2.2)
Median	6	6	6
Min/max	1/12	1/9.5	1/12
TSUI scale			
Mean (SD)	12.6 (2.9)	13.7 (3.0)	13.2 (3.0)
Median	12	13.5	13
Min/max	9/19	9/19	9/19
HADS-anxiety			
Mean (SD)	7.9 (4.2)	7.6 (4.4)	7.7 (4.3)
Median	8	6.5	8
Min/max	0/17	0/16	0/17
HADS-depression			
Mean (SD)	8.3 (3.7)	8.5 (3.9)	8.5 (3.8)
Median	9	8.5	9
Min/max	0/13	2/17	0/17
OnabotulinumtoxinA dose			
Mean (SD)	247.2 (35.1)	257.4 (40.1)	252.6 (37.9)
Median	250	265	257.5
Min/max	165/300	150/320	150/320

### Efficacy

**Fig. 2:**
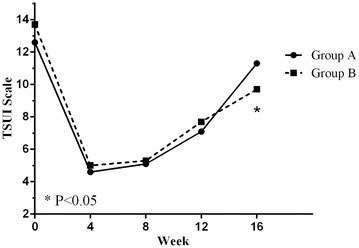
Changes in Tsui score

No significant differences were observed in the Tsui score between group A and group B at 4, 8, and 12 weeks after treatment (p > 0.05). However, 16 weeks post-treatment, the Tsui score in group A was significantly higher than that in group B (p < 0.05) (Fig. 2).

The pain associated with CD at baseline was similar in both groups, with 12/32 (37.5 %) group A patients and 14/36 (38.9 %) group B patients being pain-free. No significant difference in degree of pain was observed between the two groups at baseline or at any time point during follow-up (p > 0.05) (Table 2), although for both groups, the degree of pain at each time point significantly reduced compared to that at pre-treatment conditions (p < 0.05).

**Table 2:** Neck pain as measured by VAS, mean (SD)

	Group A	Group B	Total
Baseline	3.0 (3.1)	3.3 (3.2)	3.2 (3.1)
Week 4	1.0 (1.4)	0.7 (1.2)	0.8 (1.3)
Week 8	1.1 (1.6)	0.9 (1.4)	1.0 (1.5)
Week 12	1.4 (1.6)	1.3 (1.3)	1.3 (1.5)
Week 16	1.9 (2.1)	2.5 (2.4)	2.1 (2.3)

No significant difference in HADS score was observed between group A and group B 8 weeks post-treatment (p > 0.05). Varying degrees of anxiety occurred in 54.4 % (37/68) of patients, and varying degrees of depression were observed in 64.7 % (44/68) of patients. The HADS scores decreased at 8 weeks post-botulinum toxin type A treatment, but this difference was not significant (p > 0.05).

Using the patient-rated PGIC, 59.3 % (19/32) of patients in group A rated their overall condition as “very much improved” or “much improved,” whereas 61.1 % (22/36) of patients in group B rated their condition as “very much improved” or “much improved” 16 weeks after treatment. There was no significant difference in PGIC findings between groups (p > 0.05). The CGIC findings paralleled the PGIC results. However, compared to PGIC findings, higher percentages of patients, 71.9 % (23/32) and 72.2 % (26/36), respectively, were rated “very much improved” or “much improved.” No significant difference was observed between groups. In both groups, a significantly higher proportion of patients “very much improved” or “much improved” using the CGIC than using the PGIC (p < 0.05).

### Safety

Adverse reactions occurred in 53.1 % (17/32) and 52.8 % (19/36) of patients in group A and group B after treatment, respectively. However, the incidence of dysphagia in group A was significantly higher than that in group B (p < 0.05). A significantly higher incidence of discomfort at the injection site occurred in group B, than that in group A (p < 0.05) (Table 3). All adverse events occurred within 2 weeks and completely disappeared within 8 weeks. Adverse reactions during the trial were very mild, and no special treatment was required.

**Table 3:** Summary of adverse events, N (%) of patients

Adverse events	Group A	Group B
Dysphagia	10 (31.3 %)	6 (16.7 %)
Neck muscle weakness	5 (15.6)	6 (16.7 %)
Discomfort at injection site	3 (9.4 %)	7 (19.4)
Dry mouth	2 (6.3 %)	3 (8.3 %)
Patients with ≥1 adverse events	17 (53.1 %)	19 (52.8 %)

One patient in group A and 2 patients in group B discontinued the study owing to lack of efficacy or lack of follow-up.

## Discussion

Botulinum toxin type A inhibits peripheral acetylcholine release by cleaving synaptosomal-associated proteins of 25 kDa (SNAP-25) in the presynaptic membrane of cholinergic nerve terminals, inducing chemical denervation of muscles and relieving symptoms of dystonia. After botulinum toxin type A injection, new motor end plates are formed by the sprouting of nerve terminals. Therefore, long-term and repeated botulinum toxin type A injection is required for the treatment of CD, making it crucial to compare the efficacies of various injection techniques. There are several common injection techniques for botulinum toxin type A, including the use of palpation, EMG, electrical stimulation, and ultrasound guidance.

Despite controversies regarding the use of botulinum toxin type A treatment for dystonia, the majority of studies indicate that injection guided by palpation and EMG result in comparable outcomes. In the present study, the efficacy and safety of botulinum toxin type A injection guided by the two techniques, palpation and EMG, were compared in the treatment of CD patients. In agreement with previous findings (Truong et al. 2005; Hallett et al. 2013), clinical symptoms of patients significantly improved following botulinum toxin type A injection, and the Tsui score significantly decreased compared to baseline. Few studies have evaluated the effect of injection techniques on CD treatment. Comella et al. (1992) found that EMG assistance did not further increase the number of patients improving compared with those injected clinically. The striking difference between two group was the increased number of patients with marked improvement as well as the greater magnitude of improvement in those patients injected with EMG assistance. Currently, the efficacy of various injection techniques is evaluated within 12 weeks following treatment. In the present study, no significant difference was observed in the Tsui score between groups at 12 weeks post-treatment. However, the Tsui score was higher in patients receiving palpation-guided injections than that in patients receiving EMG-guided injections 16 weeks after treatment. This may be owing to more accurate identification of spasmodic muscles facilitated by EMG-guided injection, which allows more drugs at the same dose to act on the motor end plates, thereby resulting in prolonged efficacy. However, whether EMG-guided injection can prolong efficacy duration requires further investigation.

In the present study, the effect of injections using two techniques on neck pain in CD was studied. Our results showed no significant difference in pain between the two injection techniques at each time point during follow-up. Pain relief was not parallel to the improvement in clinical symptoms. While a significant difference in Tsui score was observed between groups 16 weeks after therapy, no significant difference in VAS scores was observed. Consistent with previous studies, the majority of CD patients presented with symptoms of neck pain. It has been reported that neck pain was significantly relieved in majority of patients with dystonia after botulinum toxin type A therapy (Brin et al. 1988). A study using a masticatory muscle model demonstrated that botulinum toxin type A can relieve pain caused by muscle spasm, and that the analgesic effect remains when muscle tension is restored (Freund and Schwartz 2003). In addition, botulinum toxin type A exerts a significant effect on various pain disorders, including trigeminal neuralgia (Wu et al. 2012) and migraines (Aurora et al. 2010; Diener et al. 2010). However, the mechanism by which botulinum toxin type A relieves pain remains unclear. Its efficacy in both spasmodic and non-spasmodic pain suggests that the effectiveness of botulinum toxin type A in neck pain relief in CD patients might not be associated with the mechanism of dystonia alleviation. Studies have shown that the effectiveness of botulinum toxin type A in neck pain relief may be associated with its inhibitory effects on meningeal nociceptors, which prevents or reverses C-fiber-mediated mechanical hypersensitivity (Schulte and May 2015).

Previous studies have shown that CD significantly affects patient quality of life (Comella and Bhatia 2015). In the present study, more than half of the patients presented with anxiety and depressive disorders, which may affect quality of life. Our results demonstrated that the HADS score 8 weeks after treatment was not significantly different from that prior to treatment between groups, even though clinical symptoms significantly improved. These results partly explain the higher proportion of “very much improved” or “much improved” ratings with CGIC, than that observed using PGIC, since ratings may be distorted owing to self-evaluation. These findings suggest that attention is required in clinical practice to simultaneously treat anxiety and depression associated with CD, thereby achieving optimal outcomes.

Botulinum toxin type A was generally well tolerated in this study population. The incidence of dysphagia was significantly lower, whereas the incidence of discomfort at the injection site was significantly higher in the group of patients receiving EMG-guided injection. These results may have been owing to increased accuracy of injection facilitated by EMG guidance, thereby resulting in less diffusion of the drug to the muscles of the throat (Werdelin et al. 2011). Compared with palpation-guided injection, EMG-guided injection requires more complicated procedures, in which repeated adjustment of the needle electrode position may result in discomfort at the injection site in patients. Because doses of different formulations of Botulinum toxin type A are not interchangeable, it is not clear whether other formulates of Botulinum toxin type A can exert the same effects. Although we found that 1U of the Botulinum toxin type A we used herein corresponds to 1 U of commercially available Botox (Allergan, Inc.) in the treatment of blepharospasm and hemifacial spasm (Wu et al. 2011), more studies are needed to evaluate the effective dose of other formulations of Botulinum toxin type A in the management of CD (Wu et al. 2012).

In summary, our 16-week follow-up, randomized, controlled trial indicated that CD patients treated with EMG guidance experienced a prolonged benefit as measured by the Tsui scale when compared to CD patients treated with palpation guidance alone. CD patients treated with EMG guidance experienced a significantly more pain at the injection site but a significantly lower adverse event occurrence rate of dysphagia when compared to CD patients treated with palpation guidance only.
